# Evaluation of postural stability and vestibulo-ocular reflex in adults with chronic suppurative otitis media

**DOI:** 10.1007/s00405-022-07687-y

**Published:** 2022-10-27

**Authors:** HebatAllah Abdelmotaleb, Ossama Sobhy, Mohamed Bassiouny, Mayada Elsherif

**Affiliations:** 1 Audio-Vestibular Medicine Unit, Department of Otorhinolaryngology, Faculty of Medicine, Alexandria University, Chamblion Street, El Azareeta, Alexandria, Egypt; 2 Department of Otorhinolaryngology, Faculty of Medicine, Alexandria University, Alexandria, Egypt

**Keywords:** Chronic suppurative otitis media, Vestibular dysfunction, Posturography, Video head impulse test, Conductive hearing loss, Mixed hearing loss

## Abstract

**Purpose:**

To evaluate the vestibulospinal reflex and vestibulo-ocular reflex (VOR) in patients with chronic suppurative otitis media (CSOM) using posturography and the video head impulse test (vHIT).

**Methods:**

Sixty-five patients with CSOM and 65 healthy participants as controls were included. Patients with CSOM were instructed to complete the dizziness handicap inventory (DHI). All participants underwent otoscopy, pure-tone audiometry, posturography sensory organization test (SOT), and vHIT.

**Results:**

Patients with CSOM exhibited a high prevalence of dizziness. The CSOM group had poor SOT vestibular scores compared to the control group. Patients with CSOM had worse sways in the antero-posterior and mediolateral planes. The CSOM group was divided into two subgroups according to the type of hearing loss. SOT vestibular scores were significantly poorer in the mixed hearing loss group than those in the conductive hearing loss group. We found a positive correlation between disease duration and poor SOT vestibular scores. Moreover, poor SOT vestibular scores correlated with high DHI scores. We found abnormalities in the vHIT results in the CSOM group in the form of low VOR gain and corrective saccades.

**Conclusion:**

Our study provides clinical evidence of dizziness, poor postural control, and VOR abnormalities in patients with CSOM. The presence of sensory elements of hearing loss in patients with CSOM appears to be positively associated with vestibular dysfunction.

## Introduction

Chronic suppurative otitis media (CSOM) is one of the most prevalent chronic infectious diseases, particularly in developing countries [1]. It is a significant cause of hearing loss and can cause extra- and intracranial complications [2]. Many long-term consequences of CSOM such as sensorineural hearing loss (SNHL), tinnitus, psychological challenges, and poor quality of life have been reported [3, 4]. Traditionally, the term CSOM is defined as “chronic inflammation of the middle ear and mastoid cavity, which presents with recurrent purulent otorrhea through tympanic membrane perforation [5]”.

Structural damage to the cochlea and vestibule owing to CSOM has been widely demonstrated [6–8]. Studies have shown that bacterial products and inflammatory mediators may spread through the round window membrane to the labyrinth. Bacterial products and inflammatory mediators lead to the deformed expression of proteins, leading to injury and damage to the cochlea and vestibule. The loss of hair cells occurs because of toxins and enzymes absorbed from the large intercellular spaces in the round window [9, 10]. However, the clinical effects of cochlear and vestibular damage due to CSOM are variable and have not been widely reported. SNHL and dizziness are reported to result from such damage [3, 11].

Abnormalities in the results of the caloric test, rotary chair test, and vestibular evoked myogenic potentials (VEMP) have been reported in CSOM. However, vestibular test findings do not correlate well with patients’ clinical symptoms of dizziness [11–13]. Clinical evidence suggests that patients with chronic otitis media (COM) have a high probability of having vestibular dysfunction, which can negatively influence their quality of life [11, 14–16]. The diagnosis of vestibular dysfunction in CSOM patients is often frustrating because the use of tap water in the caloric test is contraindicated in perforated tympanic membranes and substituted by an air caloric test [17]. Similarly, videonystagmography and VEMPs have certain limitations and are affected by the middle ear status [18, 19].

The peripheral vestibular system consists of the macula organs (utricle and saccule) and three semicircular canals (SCCs), which are connected to neural pathways. The function of the vestibular system includes the sensation of orientation and acceleration of the head in any direction, with associated compensation in posture and eye movement. These reflexes are referred to as the vestibulospinal reflex (VSR) and vestibulo-ocular reflex (VOR), respectively [20]. Currently, posturography and video head impulse test (vHIT) are important constituents of functional investigations to identify and assess postural stability and VOR, respectively.

Posturography is an objective tool that is used to assess postural stability. It is not influenced by the subjective interpretation. The results of the posturography are illustrated graphically and documented numerically. Static posturography is performed by making the participant stand on a fixed platform, connected to detectors, and measuring the minute sways of the body. Dynamic posturography involves perturbing the participant’s posture using a foam cushion or a movable horizontal and tilting platform [21]. The vHIT is a commercially available tool that enables clinicians to examine the VOR of each SCC individually; however, it assesses high-frequency (4–5 Hz) responses [22, 23]. VOR is impaired by gain reduction and the presence of corrective saccades [24]. Herein, we evaluated vestibular function in patients with CSOM using posturography and vHIT to assess the VSR and VOR, respectively, in a simple and functional manner.

## Materials and methods

### Design, ethical considerations, and participants

This comparative study was conducted at a tertiary-care medical center. It included 65 adults with CSOM and 65 matched healthy controls (matched in age, sex, and height), and it was conducted in outpatient clinics from September 2020 to October 2021. Ethical approval was obtained from the Research Ethics Commission of our institution (IRB NO:00012098-FWA NO:00018699), and it conformed with the principles of the Declaration of Helsinki and the Belmont Report. Informed consent was obtained from all participants.

We selected patients diagnosed with unilateral or bilateral CSOM**,** with ages ranging from 20 to 60 years**,** who could stand independently in an upright position, and could establish all study requirements. We excluded patients with ear surgical interventions; history of ototoxic drug administration or currently on vestibular sedatives; any other peripheral vestibular and otological pathology; any central cause of vestibular dysfunction; severe visual impairment; arthritic or orthopedic conditions affecting the ankles, knees, hips, or back; musculoskeletal and neurological abnormalities; and abnormal proprioceptive functions such as diabetes mellitus.

## Methods

A complete medical history was obtained from each patient, and all available medical records and investigations were reviewed. A thorough physical clinical examination was performed. Otoscopy was conducted for inspection of the external auditory canal, tympanic membrane perforation, discharge, and middle ear.

### Dizziness handicap inventory (DHI)

Each patient was instructed to complete the DHI, a self-report questionnaire comprising 25 items. The DHI was developed to assess the handicapping and disabling effects of dizziness. This self-assessment measures the functional, emotional, and physical effects of dizziness in individuals aged ≥ 19 years. The DHI was completed in 5–10 min [25]. Each question was answered with either Yes, Sometimes, or No. The DHI was scored as follows: Yes = 4, Sometimes = 2, and No = 0. The highest possible score is 100, which indicates the maximum perceived disability, while the lowest score is 0, indicating no perceived disability. Higher scores signify a greater perception of handicap owing to dizziness [26]. We used the Arabic version of the DHI [27].

### Pure-tone audiometry

Pure-tone audiometry was performed on all participants to evaluate the degree and type of hearing loss. The test was performed in a sound-proof room using an AD629 audiometer (Interacoustics, Assens, Denmark). Air conduction thresholds were measured using TDH39 headphones. Bone conduction thresholds were measured using the mastoid placement of the bone vibrator.

### Posturography

All participants underwent sensory organization test (SOT) of posturography using the Synapsys Posturography System (SYNAPSYS, Marseille, France). A static platform was used to perform static posturography. Dynamic posturography was performed using a foam platform that imposed a dynamic balance task. The foam platform was positioned on a static platform. Six conditions were created: eyes open (EO), eyes closed (EC), and vision erroneous (VE) trials were performed on the static and dynamic support surfaces. These are:

1. Static support, EO: none of the afferents are altered.
2. Static support, EC: the visual inputs are suppressed. The somatosensory and vestibular inputs are not altered.
3. Static support, VE: the visual information is inaccurate through the “sway referencing” of the visual surroundings. The somatosensory and vestibular inputs are not altered.
4. Dynamic support, EO: the somatosensory information provided from the feet and joints is erroneous; the visual and vestibular inputs are not altered.
5. Dynamic support, EC: the somatosensory information is erroneous; the visual inputs are suppressed. The vestibular inputs are not altered.
6. Dynamic support, VE: the somatosensory and visual inputs are erroneous; the vestibular inputs are not altered.

In each test condition, the participants were instructed to stand straight without stiffness on the support surface, without moving their bare feet. The participants were instructed to look at a figure on a screen in front of them and were instructed to maintain equilibrium. Two trials of 20 s duration were recorded for each test condition. During each trial, the center of gravity (COG) sway angle was computed in real-time based on the biomechanical relations between the positions of the center of vertical force, COG, and limits of stability. Real-time, multiple-pole complex digital filters were used to approximate these amplitude and frequency relations.

The equilibrium score (ES), a measure of stability, was computed for each trial. ES is a non-dimensional percentage that compares the patient’s peak amplitude of antero-posterior (AP) and mediolateral (ML) sways to the theoretical AP and ML limits of a normal person of the same age and height. An ES near 100% indicates little sway (perfect stability), while scores near zero indicate loss of balance. For example in the 5th condition (dynamic support, EC), in which somatosensory input is erroneous by foam and the visual input is eliminated by the visual condition “eyes closed,” the ES score represents the patient’s ability to use his vestibular system.

The participant’s ability to use inputs from the somatosensory, visual, or vestibular systems to maintain balance is calculated by dividing the score in conditions 2, 4, or 5 by the score in condition 1, respectively, and that is called the sensory analysis ratio. For example, the sensory analysis ratio of vestibular function calculated by the device using the following equation: vestibular score = (dynamic support, EC)/(static support, EO), which represents the ratio between the condition 5 in which somatosensory and visual inputs are manipulated and the first condition in which the three balance inputs (somatosensory, visual, and vestibular) are not manipulated. The vestibular score is presented as a percentage. The vestibular score was considered “abnormal” when it was lower than the normative data provided by the posturography manufacturer. An abnormally low vestibular ratio indicated that patients made poor use of vestibular inputs for postural stability [28].

### Video head impulse

All participants underwent vHIT using the ICS Impulse vHIT system (GN Otometrics, Taastrup, Denmark). The tests were performed by the same physician. Trials with artifacts were eliminated and repeated. The participants wore tightly fitted goggles that were equipped with a high-velocity camera and mirror reflecting the right eye. Nine axis motion sensors were placed in the goggles to measure head movement in space. The participants were seated 1 m from the target, which was a dot on the front wall [29]. For the horizontal SCC examination, the participant’s head was tilted forward by 30°, and the participant was instructed to look directly at the target. Sudden and unpredictable head impulses to the right or left at 10–20° were performed. We used Migliaccio et al. modified procedure to assess vertical SCCs [30]. The participant’s head was rotated 30–40° to the right in the left anterior right posterior test or to the left in the right anterior left posterior test while fixating gaze on the target at the corner of the eye. Unpredictable forward and backward head thrusts of 10–15° were performed in the plane of the vertical canals that allowed stimulation of the anterior and posterior SCC, respectively. Ten accepted head impulses were applied in each direction for each plane. VOR was impaired by gain reduction and the presence of corrective saccades.

### Statistical procedures

The IBM SPSS software package version 20.0 (IBM Corp., Armonk, NY) was used for statistical analyses. The Kolmogorov–Smirnov test was used to assess the normality of the data distribution. The chi-square test was used for comparisons between categorical variables. The Student’s *t* test was used to compare two independent quantitative variables for parametric data, and the Mann–Whitney test was used for non-parametric data. We used Spearman’s rank correlation for non-parametric data. The significance of the results was determined at the 5% level.

## Results

### Demographic data and descriptive statistics

**Fig. 1:**
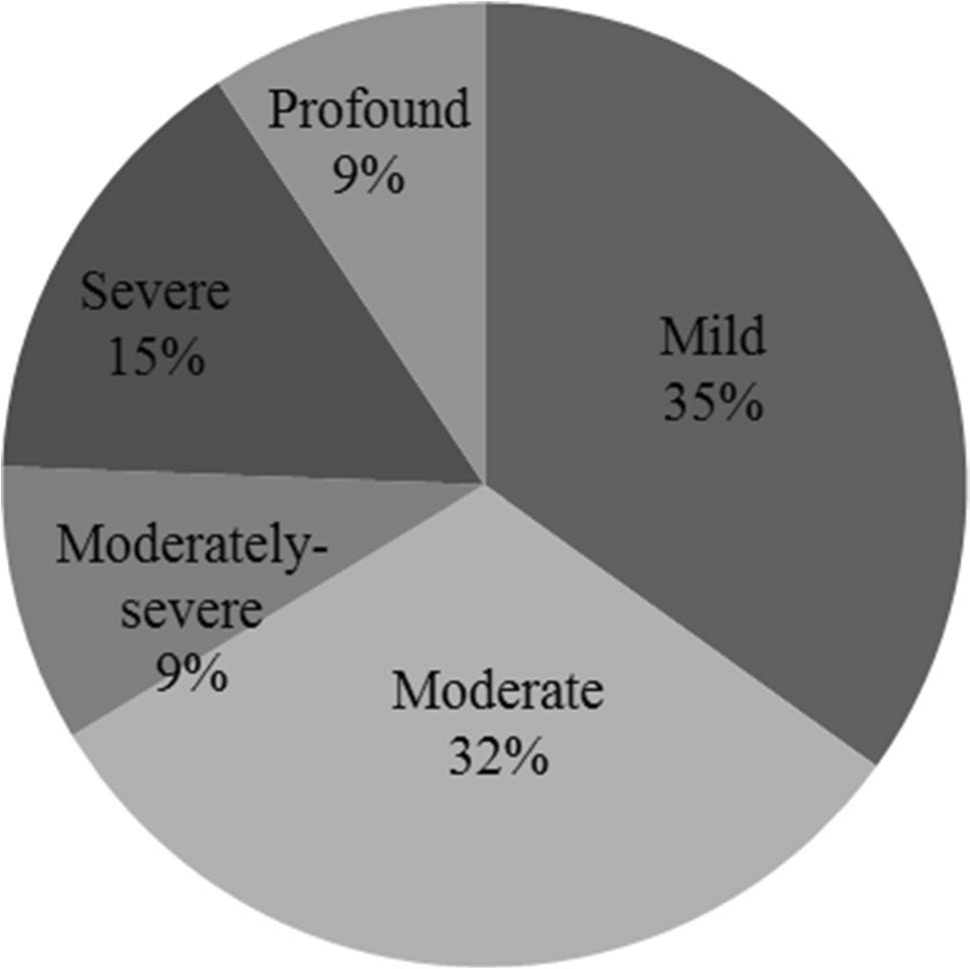
Pie chart showing the distribution of the patients with chronic suppurative otitis media (CSOM) according to the degrees of hearing loss

This study included 65 adult patients with CSOM and 65 matched healthy controls. The demographic information is listed in Table 1. The CSOM group comprised 41 (63.1%) patients with conductive hearing loss (CHL) and 24 (36.9%) with mixed hearing loss (MHL). Forty-four (67.7%) patients had unilateral CSOM and 21 (32.3%) had bilateral CSOM. In the unilateral group, the left side was affected in 31 (70.5%) patients, and the right side was affected in 13 (29.5%) patients. Figure 1 shows the distribution of CSOM according to the degree of hearing loss. All controls had normal air and bone hearing thresholds and no air-bone gaps. Thirty-four (52.3%) patients with CSOM complained of dizziness and handicap, as quantified by DHI scores. None of the participants in the control group reported dizziness.

**Table 1:** Demographic data

Demographic data	CSOM (*n* = 65)		Control (*n* = 65)		Test of sig.	*p*
	No	**%**	No	**%**		
Sex						
Male	23	35.4	25	38.5	*χ*^2^ = 0.132	0.716
Female	42	64.6	40	61.5		
Age (years)						
Min.–Max.	20.0 – 59.0		23.0 – 60.0		*t* = 1.238	0.218
Mean ± SD	38.29 ± 10.93		40.69 ± 11.17			

CSOM, chronic suppurative otitis media; *χ*^2^, *χ*^2^ for Chi square test for comparing between the two groups, SD, standard deviation; *t*, Student *t* test; *p*, *p* value for comparing between the two studied groups

### Posturography results

There was no significant difference between CSOM and the control group in somatosensory and visual scores of SOT sensory analysis ratios (*p* > 0.05). However, according to the normative data provided by the manufacturer, 9 (13.8%) and 6 (9.2%) of CSOM patients had abnormal AP and ML somatosensory scores, respectively. Seven (10.7%) and 6 (9.2%) of CSOM patients had abnormal AP and ML visual scores, respectively. All patients with abnormal somatosensory or visual scores already had abnormal vestibular scores. In the control group, none of the participants had an abnormal vestibular or visual score, but two (3.1%) had abnormal AP somatosensory scores despite the absence of an identifiable cause of imbalance.

**Fig. 2:**
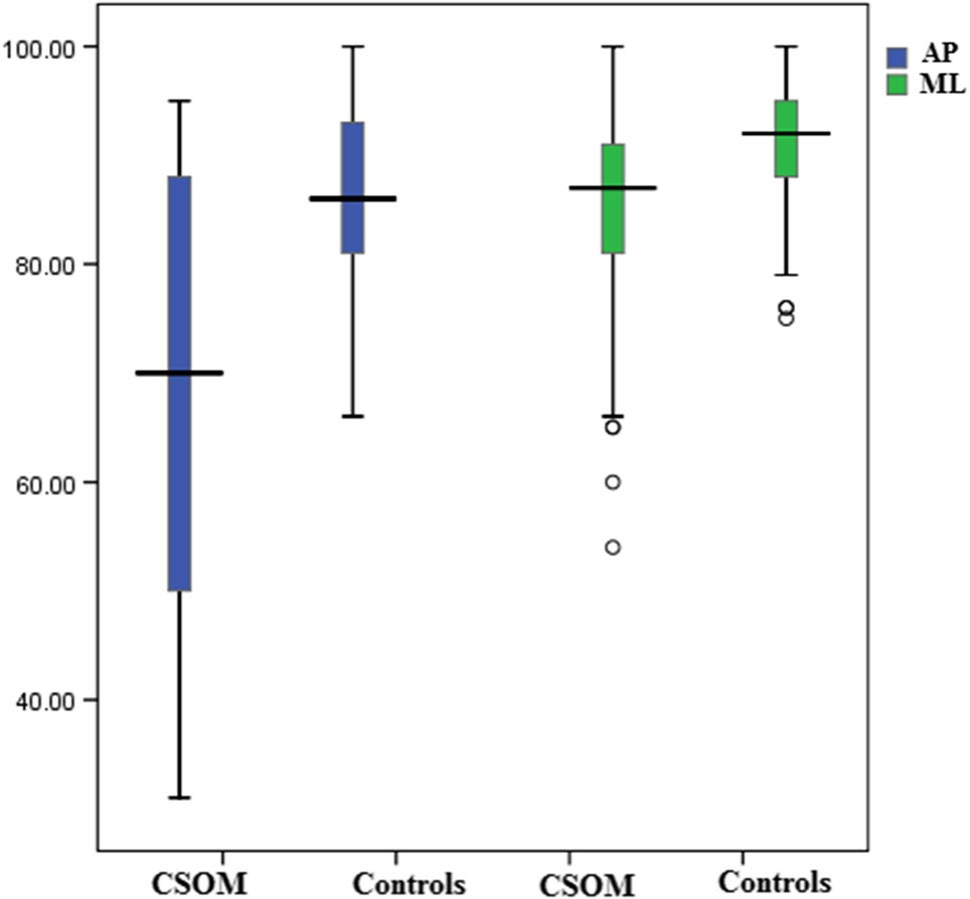
Boxplot showing comparative analysis of posturography vestibular scores between chronic suppurative otitis media (CSOM) patient and control groups in antero-posterior (AP) and mediolateral (ML) planes

**Fig. 3:**
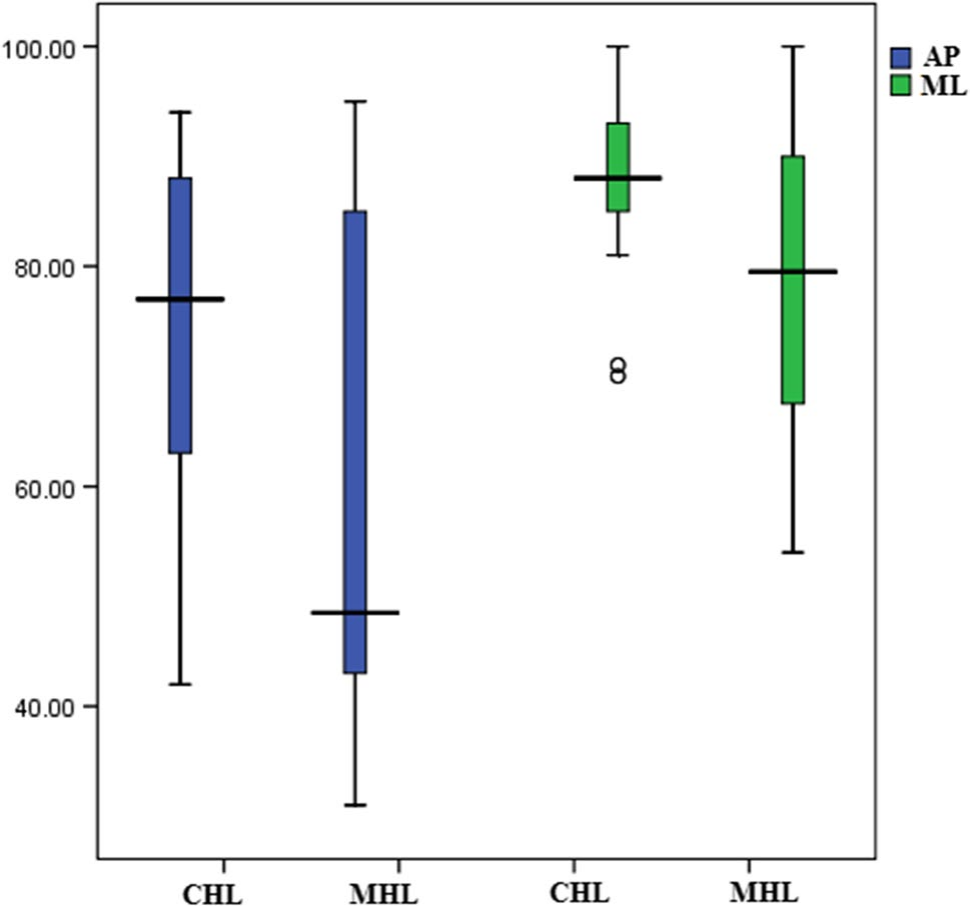
Boxplot showing comparative analysis of posturography vestibular scores between conductive hearing loss (CHL) and mixed hearing loss (MHL) groups in antero-posterior (AP) and mediolateral (ML) planes

The vestibular score of SOT showed that the CSOM group had worse sways in the AP and ML planes than those of the control group (Table 2 and Fig. 2) (*p* < 0.05). Twenty-four (36.9%) and 16 (24.6%) patients had abnormal AP and ML vestibular scores, respectively. None of the participants in the control group showed abnormal vestibular score. On comparing patients with CHL (*n* = 41) to those with MHL (*n* = 24), a statistically significant difference was found between the two groups, and the MHL group had worse vestibular scores in the AP and ML planes (*p* < 0.05) (Table 3 and Fig. 3).

**Table 2:** Comparison of the vestibular score of sensory organization test (SOT) between CSOM and control groups

	COSM (*n* = 65)	Control (*n* = 65)	*U*	*p*
AP vestibular score				
Min.–Max.	31.0–95.0	66.0–100.0	1001*	< 0.001*
Mean ± SD	69.03 ± 18.91	86.40 ± 8.14		
Median	70.0	86.0		
ML vestibular score				
Min.–Max.	54.0–100.0	75.0–100.0	1380*	0.001*
Mean ± SD	84.43 ± 10.89	90.54 ± 6.45		
Median	87.0	92.0		

*CSOM* chronic suppurative otitis media, *AP* antero-posterior, *ML* mediolateral, *SD* standard deviation

**Table 3:** Comparison of the vestibular score of sensory organization test (SOT) between the CHL and MHL groups

	CHL (*n* = 41)	MHL (*n* = 24)	*U*	*p*
AP vestibular score				
Min.–Max.	42.0–94.0	31.0–95.0	304*	0.011*
Mean ± SD	74.24 ± 14.4	60.12 ± 22.4		
Median	77.0	48.5		
ML vestibular score				
Min.–Max.	70.0–100.0	54.0–100.0	340*	0.039*
Mean ± SD	87.65 ± 7.49	78.91 ± 13.48		
Median	88	79.5		

*AP* antero-posterior, *ML* mediolateral, *CHL* conductive hearing loss, *MHL* mixed hearing loss, *SD* standard deviation

By comparing the SOT vestibular score results between bilateral and unilateral CSOM cases (*n* = 21 and 44, respectively), we found a significant difference between the two groups in the AP plane only (*p* < 0.05) and not in the ML plane. Patients with bilateral CSOM had worse sway in the AP plane.

Among the CSOM group, using Spearman’s rank correlation, a significant negative correlation was found between the duration of the disease and vestibular score (AP, *p* = 0.001; ML, *p* = 0.02). This indicates worse results associated with a longer duration of the disease. A significant negative correlation was detected between DHI scores and vestibular scores (AP, *p* < 0.001; ML, *p* < 0.001). This indicated that the posturography results reflected the patients’ clinical symptoms of dizziness. However, we found no significant correlation between air–bone gap or age and vestibular scores results ([AP, *p* = 0.74; ML, *p* = 0.16] [AP, *p* = 0.18; ML, *p* = 0.37], respectively).

### vHIT results

We did not find significant differences between the cases and controls in the mean VOR gain of the anterior, posterior, and right lateral SCCs. However, we found a significant difference between the cases and controls in the mean VOR gain of the left lateral SCC (Table 4). The mean VOR gain of the left lateral SCC in the CSOM group was significantly lower than that of the control group. We found corrective saccades and low VOR gain in at least one SCC in 10 patients (15.4%). Five patients among them had MHL. They represented 20.8% of patients in the MHL group (*n* = 24). Five patients had lateral SCC saccades, three had posterior SCC saccades, one had lateral and posterior SCC saccades, and one patient had anterior and lateral SCC saccades. Two lateral and one posterior SCCs saccades were overt saccades; otherwise, they were covert saccades. No participants in the control group had a low VOR gain or saccades.

**Table 4:** Comparison of mean vestibulo-ocular reflex (VOR) gain between CSOM and control groups

	CSOM (*n* = 65)	Control (*n* = 65)	*t*	*p*
Lt. Lat.				
Mean ± SD	0.91 ± 0.15	0.98 ± 0.10	2.825*	0.006*
Rt. Lat.				
Mean ± SD	0.96 ± 0.13	1 ± 0.11	1.9	0.058
LA				
Mean ± SD	0.94 ± 0.15	0.96 ± 0.15	0.774	0.441
RP				
Mean ± SD	0.90 ± 0.14	0.93 ± 0.14	1.07	0.28
LP				
Mean ± SD	0.89 ± 0.17	0.92 ± 0.14	0.959	0.34
RA				
Mean ± SD	0.95 ± 0.13	0.97 ± 0.16	0.81	0.416

*CSOM* chronic suppurative otitis media, *Lt. Lat.* left lateral, *Rt. Lat.* right lateral, *LA* left anterior, *RP* right posterior, *LP* left posterior, *RA* right anterior, *SD* standard deviation

## Discussion

This comparative study was conducted to detect vestibular dysfunction in adult patients with CSOM using computerized posturography and vHIT. The study also questioned whether there was a link between cochlear and vestibular dysfunction.

Various studies have shown the presence of vestibular dysfunction in CSOM [11, 12, 31, 32]. We used posturography in our study because posturography is a useful preliminary diagnostic test for detecting peripheral vestibular damage [33]. Posturography evaluates balance, including the VSR. Balance does not depend only on vestibular inputs and VSR but also on other sensory inputs (somatosensory and visual) and the motor control system. Posturography is not considered a direct tool for the evaluation of vestibular function. However, it provides functional information regarding how well an individual can use balance in daily activities. Studies have been done to know which parts of the peripheral vestibular end organ are reflected by posturography. Fujimoto et al. demonstrated that foam posturography is correlated with cervical VEMPs (cVEMP) and concluded that damage to the saccule and inferior vestibular nerve system could affect postural stability [34]. ‏ Studies demonstrated that foam posturography correlated with ocular VEMPs (oVEMP), suggesting that foam posturography may reflect utricular function [35, 36]. Liu et al. studied the correlation between the vestibular score of SOT and VEMP results. They found that oVEMP exerted the greatest effect on the vestibular score, followed by age and cVEMP. They concluded that SOT could mainly reflect utricular function [36].

In our study, we found no significant difference between the two groups in SOT somatosensory and visual scores. However, there were some patients in the CSOM group had abnormal somatosensory and visual scores despite the strict limitations in our selection of participants. All patients with abnormal somatosensory or visual scores had no apparent cause of imbalance. In addition, they already had abnormal vestibular scores. Maybe the vestibular dysfunction overrides the other sensory inputs.

We found that 36.9% of the patients in the CSOM group had abnormal SOT vestibular scores, and the scores of the CSOM group were significantly poorer than those of the control group. In accordance with our study, Monsanto et al. found abnormal findings of posturography in patients with CSOM [31]. However, Mostafa et al. identified normal response patterns on posturography despite their abnormal findings in VEMP, rotatory chair, and caloric tests, and their study did not include healthy controls for comparison [12].

In our study, the SOT vestibular scores of patients with MHL were significantly lower than those of patients with CHL. This indicates that the pathology affects both the cochlea and vestibule. Pathological studies by Cureoglu et al. and Joglekar et al. found damage to the outer and inner hair cells in the basal turn of the cochlea in patients with COM in comparison with their normal ears. They found that in the basal turn, the stria vascularis and spiral ligament were reduced in the area of the ears with COM [8, 37]. Cochlear damage is consistent with the prevalence of SNHL in CSOM [38]. Monsanto et al. demonstrated more massive damage in the late stages of COM. The damage appeared to initiate from the basal to the middle turn of the cochlea, and then to the saccule and utricle[7]. However, spiral and Scarpa ganglion cells were intact [39]. These studies indicated that both cochlear and vestibular sensory cells were affected by COM, explaining the finding in our study that SNHL is associated with increased vestibular dysfunction.

We found that patients with bilateral CSOM had worse posturography results in the AP plane than the unilateral cases. This may be due to the chronic and slow progression of the disease, which allows for better compensation in unilateral cases. Studies have shown that patients with bilateral vestibulopathy are either unable to maintain (or have reduced) balance compared to patients with unilateral vestibulopathy [40].

In our study, 52.3% of patients complained of dizziness. Our results are consistent with those of previous studies [12–14]. We used the DHI to quantify the severity of dizziness handicap in the patients. The DHI is considered a reliable and valid tool for assessing vestibular pathology in adults [25]. It quantifies dizziness disability to numerical values that ease correlation with posturography values. There was a negative correlation between the DHI and vestibular scores, indicating that posturography is a good diagnostic tool for dizziness in patients with CSOM. In contrast, other tools (caloric test, rotational chair, and VEMP) did not show a good correlation with clinical symptoms [12, 13]. Our study revealed a correlation between the duration of the disease and poor vestibular scores, which is consistent with clinical and histopathological studies that showed an association between a long duration of disease and severity of vestibular dysfunction [7, 12, 38].

There was no correlation between age and vestibular scores and that excluded age as a confounding factor that precipitates poor balance and/or SNHL [41, 42]. In addition, there was no correlation between the air–bone gap and vestibular scores, which can be explained by the fact that the air–bone gap is determined by the size and site of the perforation, ossicular erosion, and pneumatization of the middle ear and mastoid. The air–bone gap does not reflect the extent or severity of the inflammatory process or the possibility of membranous labyrinth affection [43].

We used the vHIT for the assessment of SCCs because it has some advantages over the air caloric test. The vHIT enables individual examination of the VOR of each SCC; however, the caloric test evaluates horizontal SCC only. In addition, the caloric test measures low-frequency responses only. Using an air caloric test in a moist ear (with either a large perforation or a cavity) can exhibit inverted caloric nystagmus in warm air caloric stimulation, while dry open mastoid or fenestration cavities may exhibit hyperactive caloric responses [17, 32]. In a study by Siampara et al., they found a low incidence of canal paresis (5.3%) in hot and cold caloric tests in CSOM patients. This is because, as mentioned before, the caloric test gives information about low-frequency responses of horizontal SCC only [38].

We found significantly reduced VOR gain in the left lateral SCC of the CSOM group compared to controls. We also found corrective saccades and a low VOR gain in 15.4% of the cases. In a study by Tomaz et al., who evaluated VOR in COM, 18% of the CSOM subgroup had corrective saccades and no significant difference in VOR gain compared with controls [44]. Another study by Monsanto et al. found no significant differences in VOR gain between the CSOM subgroup and control group; however, 23% of them had abnormal vHIT results [31]. In previous studies, there was no significant difference in VOR gain, which may be owing to the small sample size of the CSOM group compared with ours. Monsanto et al. in pathological study found a reduced density of types I and II hair cells in the lateral SSC, saccule, and utricle of the COM group. Type I hair cells are only reduced in the posterior SCC [6]. We found that the  prevalence of lateral SCC abnormalities were greater than that of vertical canals, which may be owing to the spread of inflammatory mediators and their concentrations in different parts of the membranous labyrinth.

The percentage of abnormal results in vHIT is relatively lower than the percentage of abnormal results in posturography, which can be explained by the dysfunction of macula organs (utricle and saccule), which engage in VSR, being more than SCCs that engage in VOR [7]. The aforementioned hypothesis was supported by Chang et al., who reported that vestibular dysfunction is more significant in the saccule and utricle, whereas SCC deficits occur later [45]. Moreover, vHIT evaluates the VOR at a high frequency in which only type I hair cells are involved; it is possible that damage to hair cells type II is more and that not measured by vHIT [24].

Our study has some cons. Besides posturography, the only direct test for vestibular function is the vHIT. Posturography is not considered a direct tool for evaluation of vestibular function. Posturography has been shown in studies to reflect utricular function, but it is not specific for assessing vestibular function. Posturography does not provide site, laterality, or etiology information. Posturography findings may differ from other vestibular laboratory testing because posturography takes compensation into account. We aimed to establish a clinically simple approach to diagnose dizziness in CSOM without overestimations. Unfortunately, our study does not provide direct evaluation of macula organs (utricle and saccule). We suggest including utricle and saccule specific tests in further studies, at least in patients who have dizziness, mixed hearing loss, or abnormal posturography results. oVEMP and cVEMP evaluate utricle and saccule, respectively. However, there are some technical difficulties in the implementation of bone VEMP, but it is helpful in confirming and localizing vestibular pathologies. Other clinical tests like subjective visual vertical and subjective visual horizontal tests can also be used in addition to posturography.

This study suggests the use of posturography and vHIT as diagnostic tools to evaluate dizziness in patients with CSOM. As they provide preliminary and comprehensive ideas regarding vestibular function, this is particularly true when combined with specific utricle and saccule tests to provide information about VSR, VOR, and vestibulo-colic reflex. More studies are needed in this field to establish a good diagnostic protocol for dizziness in CSOM patients. This will be useful for future management of patients with CSOM. Therefore, after the diagnosis of vestibular dysfunction in patients with CSOM, they should be involved in suitable vestibular rehabilitation programs. All of the aforementioned measures will help improve the quality of life of CSOM patients.

## Conclusions

Our study provides clinical evidence that CSOM is accompanied with dizziness, poor postural control, and VOR abnormalities. The presence of sensory hearing loss in CSOM patients appears to be positively associated with vestibular dysfunction.
